# Indole induces acute paralysis followed by compensatory stress-induced sleep

**DOI:** 10.17912/micropub.biology.001738

**Published:** 2025-08-07

**Authors:** Nikki Diya, Mercedes I. Pierce, Shantanu Bhatt, Matthew D. Nelson

**Affiliations:** 1 Biology, Saint Joseph's University, Philadelphia, Pennsylvania, United States

## Abstract

Stress-induced sleep of *Caenorhabditis elegans* occurs following exposure to noxious stressors, such as pore forming toxins, extreme temperature, ultraviolet irradiation, tissue wounding, and viral infections. Enteropathogenic *Escherichia coli* (EPEC) is a pathogenic bacterium which can infect *C. elegans* , however stress-induced sleep in response to EPEC has not been investigated. EPEC affects worms via two distinct mechanisms: 1) Contact-independent paralysis which occurs following the release of the toxin indole; 2) Contact-dependent bacterial colonization of the intestine. Here we examine mechanism one; we find that indole induced paralysis is regulated independently of the ALA and RIS sleep neurons. In fact, impairing ALA and RIS function caused animals to paralyze significantly faster than controls. Increasing cholinergic or GABAergic input, accelerated or delayed paralysis, respectively. Worms exposed to indole who were subsequently rescued to normal growth plates displayed a compensatory stress-induced sleep that was RIS but not ALA dependent. This work will allow for detailed future investigations into indole's mechanism of action, EPEC pathogenicity, and how bacterial infection leads to recovery sleep.

**Figure 1. Indole induces acute paralysis followed by compensatory RIS-dependent stress-induced sleep.**
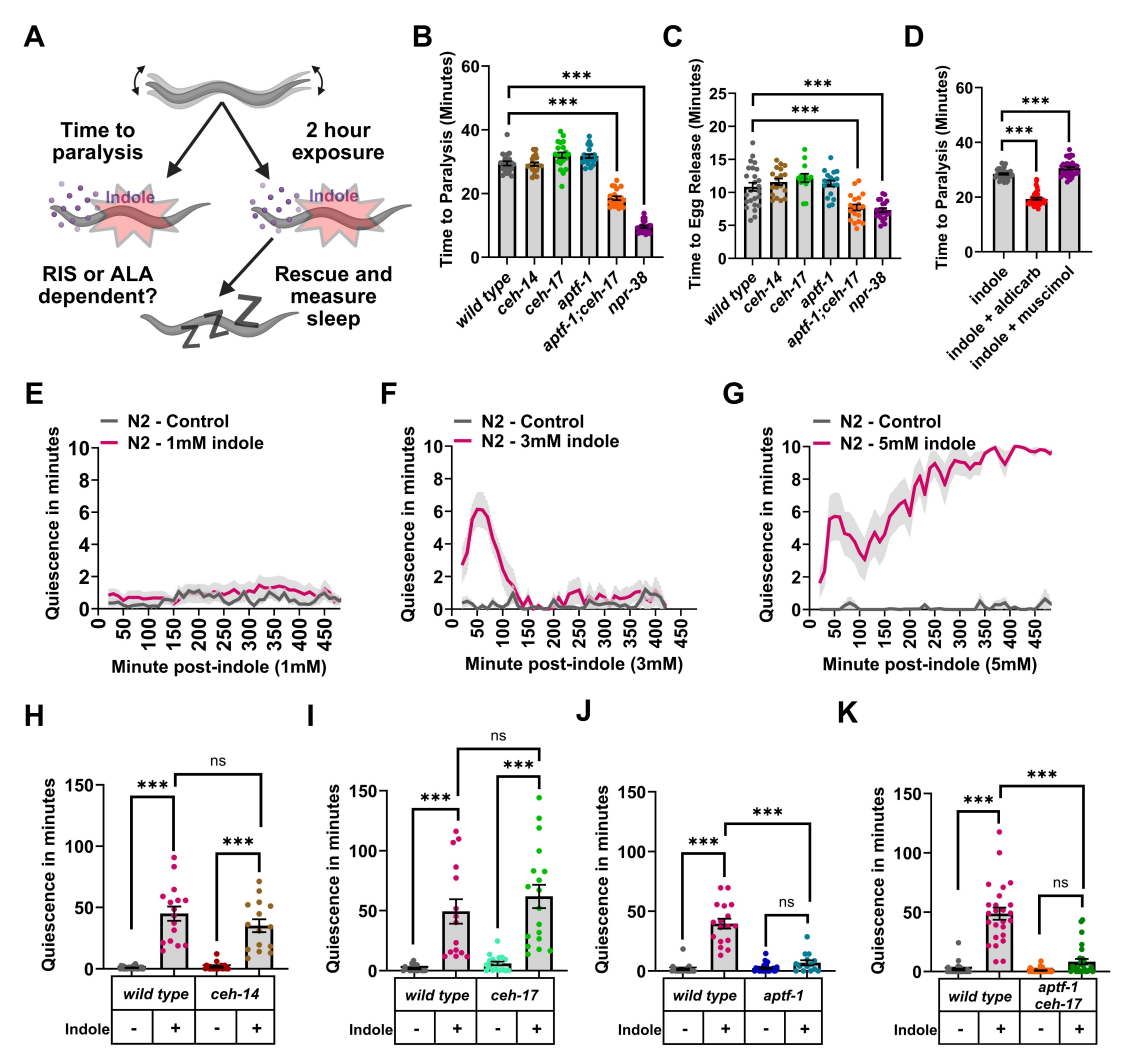
A) Schematic of the behavioral analyses. Created with BioRender.com. B) Time in minutes to paralysis on growth plates supplemented with 3mM indole in wild-type, *ceh-14* ( *ch3* ), *ceh-17* ( *np1* ), *aptf-1* ( *gk794* ), *aptf-1* ( *gk794* ); *ceh-17* ( *np1* ), and *npr-38* ( *stj367* ) animals. C) Time in minutes to egg release on growth plates supplemented with 3mM indole in in wild-type, *ceh-14* ( *ch3* ), *ceh-17* ( *np1* ), *aptf-1* ( *gk794* ), *aptf-1* ( *gk794* ); *ceh-17* ( *np1* ), and *npr-38* ( *stj367* ) animals. D) Time in minutes to paralysis on plates supplemented with indole, indole + aldicarb, or indole + muscimol, in wild-type animals. E-G) Average minutes of quiescence in 10-minute windows following a 2-hour exposure to 1mM, 3mM, or 5mM indole in wild-type animals. H) Total minutes of movement quiescence in 3 hours in controls (no indole), or following a 2-hour exposure to 3mM indole in wild-type, and *ceh-14* ( *ch3* ) animals. I) Total minutes of movement quiescence in 3 hours in controls (no indole), or following a 2-hour exposure to 3mM indole in wild-type, and *ceh-17* ( *np1* ) animals. J) Total minutes of movement quiescence in 3 hours in controls (no indole), or following a 2-hour exposure to 3mM indole in wild-type, and *aptf-1* ( *gk794* ) animals. K) Total minutes of movement quiescence in 3 hours in controls (no indole), or following a 2-hour exposure to 3mM indole in wild-type, and *aptf-1* ( *gk794* ); *ceh-17* ( *np1* ) animals. Statistical significance was calculated by one-way ANOVA following by Tukey's multiple comparisons test (N≥12, ***p<0.001).

## Description

During sickness, sleep is enhanced to promote robust immune responses for recovery (Imeri and Opp 2009). Mechanisms regulating sickness sleep are poorly understood. Model organisms have become useful tools for understanding how and why the immune system makes us sleep (Davis and Raizen 2017). In *Drosophila melanogaster* , sleep is heightened in response to bacterial infection, trauma (Kuo et al. 2010), and noxious heat (Lenz et al. 2015). In *Caenorhabditis elegans* stress-induced sleep occurs following pathogen exposure and injury, such as orsay viral infections (Iannacone et al. 2024), or noxious temperature, ethanol, hyperosmotic, or ultraviolet (UV) exposures (Hill et al. 2014; DeBardeleben et al. 2017), and wounding (Goetting et al. 2020). In each of these situations, data suggest a model in which epidermal growth factors coded by *siss-1* are released directly from damaged or infected cells (Hill et al. 2024), which then bind to EGF receptors on the ALA and RIS neurons, two sleep promoting neuropeptidergic interneurons (Van Buskirk and Sternberg 2007; Konietzka et al. 2020). Neuropeptides expressed by ALA and RIS, and multiple neuropeptide receptors, like *npr-38* (Le et al. 2023), are required for sleep, which consists of quiescence of movement, feeding, and defecation, as well as a reduction in sensory arousal (Nelson et al. 2014; Nath et al. 2016; Honer et al. 2020; Konietzka et al. 2020).

Here we investigated stress-induced sleep in response to indole toxicity. Indole is a chemical secreted by enteropathogenic *Escherichia coli* (EPEC), which paralyzes hosts, and allows the bacteria to colonize the intestine (Anyanful et al. 2005; Anyanful et al. 2009; Bhatt et al. 2011). EPEC are common causes of food and water-borne illnesses, and cause debilitating diarrhea in humans, and can kill *C. elegans* (Anyanful et al. 2009; Bhatt et al. 2011), making worms a useful tool for assessing pathogenicity of EPEC mutants (Darby et al. 2002; Anyanful et al. 2005; Anyanful et al. 2009). When worms are treated with indole exogenously, they display convulsions, paralysis, and death (Anyanful et al. 2005); here, we sought to determine if paralysis is stress-induced sleep, and the behavioral effects of acute indole exposures.

To do this we transferred worms to growth plates supplemented with indole, and assessed their behavioral responses during exposure (i.e., indole paralysis), and their long-term behavior following rescue, using the WorMotel (Churgin et al. 2017) (i.e., indole-induced sleep) ( **Figure 1A** ). Mutations in the genes *aptf-1* disrupt the function of the RIS (Turek et al. 2013), while *ceh-14* or *ceh-17* mutants show impaired development of the ALA neuron (Pujol et al. 2000; Van Buskirk and Sternberg 2010); each mutant displays reduced stress-induced sleep (Hill et al. 2014; Nath et al. 2016; Konietzka et al. 2020), although it has been reported that the *ceh-14* allele *ch3* is prone to reverting to wild type (Bayer and Hobert 2018). We quantified the time in minutes for animals to paralyze on plates supplemented with 3mM indole, in wild-type, and in *ceh-14* , *ceh-17 , aptf-1* , *ceh-17 ; aptf-1* , and *npr-38* loss-of-function animals. The *npr-38* mutants were included as an additional control considering they display significantly reduced stress-induced sleep following noxious heat or UV exposure (Le et al. 2023). First-day adults of each genotype were transferred to 3mM indole plates and observed continuously. Wild-type controls paralyzed in ~30 minutes (Video S1), which was similar to the *ceh-14 , ceh-17 ,* and *aptf-1* single mutants, indicating that the ALA and RIS neurons alone are not required for paralysis. Surprisingly, both the *ceh-17* ; *aptf-1* double mutants (ALA and RIS defective), and *npr-38* single mutants paralyzed quicker than wild-type controls ( **Figure 1B** ). In addition to paralysis, it was noted that animals expelled some of their eggs in response to indole exposure. Egg expulsion can occur when uterine and vulval muscles paralyze, and/or become hyper-contracted (Schafer 2005). We found that the time to the first egg expulsion in *ceh-14 , ceh-17 ,* and *aptf-1* mutants was not different than wild-type controls, however, *ceh-17 ; aptf-1* and *npr-38* mutants released eggs more quickly ( **Figure 1C** ). As a vehicle control, wild-type animals were grown on plates supplemented with 0.3% ethanol from the L4 stage to second-day adulthood, and no paralysis was observed (N=20). Taken together, these data suggest that sleep defective animals may be more susceptible to paralysis by indole, and that paralysis with indole is not a form of stress-induced sleep.

Paralysis phenotypes in *C. elegans* have been described, and in many cases are due to defects in neurotransmission (Brenner 1974), often at the level of the neuromuscular junction (NMJ) (Richmond and Jorgensen 1999; Richmond 2005). Acetylcholine (ACh) is released by excitatory motor neurons which stimulate muscle contractions, while inhibitory GABAergic neurons counterbalance ACh signaling (Richmond and Jorgensen 1999), thus the extent of contraction is based on a balance of cholinergic and GABAergic inputs (Richmond 2005). To determine if indole causes hypercontracted or flaccid paralysis, we enhanced cholinergic or GABAergic signaling using aldicarb, which inhibits acetylcholinesterase (Johnson and Russell 1983), or muscimol, a GABA receptor agonist (McIntire et al. 1993). Specifically, wild-type animals were transferred to plates supplemented with 3mM indole and aldicarb or muscimol, and time to paralysis was measured. Enhancing Ach signaling resulted in a more rapid paralysis, while muscimol modestly delayed it ( **Figure 1C** ). These data suggest that indole induces a hypercontracted paralysis, potentially by acting at the NMJ. Additionally, it may suggest that sleep mutants, such as the *aptf-1* ; *ceh-17* and *npr-38* animals, display heightened cholinergic activity outside of sleep, considering paralysis occurred more quickly in these backgrounds.

To measure longer-term behavioral responses to indole, we first exposed wild-type animals to 1mM, 3mM, or 5mM indole for 2 hours, and then rescued them by transferring them off indole plates and onto the agar surfaces of individual wells of polydimethylsiloxane (PDMS) microchips. Movement quiescence was then measured for 8 hours using the WorMotel (Churgin et al. 2017). We found that exposure to 1mM indole did not cause movement quiescence following rescue ( **Figure 1E** ). However, 3mM indole resulted in a temporary recovery quiescence which ended after 3 hours ( **Figure 1F** ), and 5mM exposures led to death ( **Figure 1G** ). Based on this, we sought to determine if the quiescence observed after rescue from 3mM indole was stress-induced sleep. To do this, we measured quiescence in ALA and RIS defective animals. Both the *ceh-14* and *ceh-17* single mutants displayed quiescence that was similiar to wild-type controls ( **Figure 1H, I** ). However, *aptf-1* single, and *ceh-17 ; aptf-1* double mutants showed an almost complete lack of quiescence **( Figure 1J, K).** These data suggest that *C. elegans* displays stress-induced sleep in response to acute indole exposure, and that this is regulated by the RIS neuron, while the ALA is mostly dispensable.

In summary, we find that the bacterial toxin indole causes hypercontracted paralysis in *C. elegans* and that sleep mutants may be more susceptible to these effects. Moreover, acute exposure to 3mM indole causes an RIS-dependent stress-induced sleep.

## Methods

**Worm maintenance and strains:**

Animals were maintained at 20 ^o^ C on Nematode Growth Medium (NGM) agar plates (Brenner, 1974), seeded with DA837 *E. coli* (Davis et al. 1995). First-day adult hermaphrodites were staged by selecting L4 animals the day prior to experiments.

**Preparation of test plates**

Agar plates supplemented with nematode growth medium (NGM) (USBiologocal, N1000) were made according to standard protocols (Stiernagle 2006), but supplemented with indole, aldicarb, and/or muscimol. A 1M stock of indole (>99% purity, Sigma-Aldrich, I3408) dissolved in ethanol was made fresh during the preparation of growth plates. The stock was added directly to the liquid NGM to a final concentration of 1, 3, or 5 mM, prior to pouring the plates. Aldicarb (Sigma-Aldrich, 33386) and Muscimol (Sigma-Aldrich, M1523) were dissolved in ethanol and water, respectively, and added fresh to plates at a final concentration of 50mg/L (Aldicarb), and 100mg/L (Muscimol).

**Measurements of paralysis and egg expulsion**

To quantify time to paralysis, we manually observed the animals using a stereomicroscope, every 30 seconds until the time of either egg expulsion, or paralysis, the latter being defined as a total lack of movement. The average time to paralysis was calculated and compared between multiple genotypes using one-way ANOVA followed by Tukey's multiple comparisons tests.

**Measurements of stress-induced sleep**

To quantify stress-induced sleep, We used the automated sleep tracking system called WorMotel (Churgin et al. 2017). First-day adults were placed on either, 1mM, 3mM, or 5mM indole plates (tests), or plates lacking indole (controls) for 2 hours, during which time the test animals became paralyzed and expelled their eggs. Worms were rescued by transferring them onto the agar surfaces of 24-welled polydimethylsiloxane (PDMS) chips, one worm per well. The chips were imaged every 10 seconds for 8 hours, and movement quiescence was quantified (Churgin et al. 2017). During each experiment, controls (no indole) and tests (indole), were analyzed on the same PDMS chip over multiple trials. Total quiescence over the 3-hour period following rescue from indole was compared across genotype/conditions by one-way ANOVA followed by Tukey's multiple comparisons test.

## Reagents

The following strains were used during this study: N2 (wild type, Bristol), IB16 *ceh-17* ( *np1* )I, NQ1065 *ceh-17* ( *np1* )I; *aptf-1* ( *gk794* )II, HBR227 *aptf-1* ( *gk794* )II, TB528 *ceh-14* ( *ch3* )X, SJU 378 *npr-38* ( *stj367* )X.

## Data Availability

Description: Video S1. Wild-type (N2) animals exposed to 3mM indole for 30 minutes. Resource Type: Audiovisual. DOI: https://doi.org/10.22002/8cck8-kgx67
